# Animal Intelligence

**Published:** 1891-08

**Authors:** 


### ANIMAL INTELLIGENCE.

The following cases have been authentically reported to us, or have come under our personal observation.

---

A five months old black spaniel bitch, was whipped for having torn up a feather duster, she seemed humiliated and disappeared. Shortly afterwards she reappeared with a new feather duster, which she had found in a neighboring store, and and her restored gaiety seemed to indicate, that she thought that in bringing the stolen article she had repaired all harm which she had done, and for which she recognized that she had been punished.

---

A Collie Bitch, four years old, who had never been further from home than a neighboring market town, was given to a purchaser of some cattle, and sent with the latter by railroad a distance of some seventy miles; on arrival she escaped and returned to her original home in three days.

---

A Collie, of the same family, was on the porch of the farm house at 11 A. M., when the farmer told his boy, that the cows had broken the fence and were in a meadow pasture, three quarters of a mile away and instructed him to go drive them in. An hour later, at dinner, the boy said he had forgotten to go for the cows and on leaving the house found the collie driving them to the stable. In this case the conversation was entirely between farmer and boy, and no word was addressed to the dog.

---

A Fox Terrier Bitch of our own, which had been raised from a puppy in the house, was given away three years ago and only seen from time to time. She formed a close attachment to her new master, but never forgot us. We had only seen her once in the last year, in December, but in July went to the country for a day, when she showed unbounded delight. At bed time she flew past her present master's room, her ordinary sleeping place, and was on the bed frantic with joy, until her old master had his head on the pillows, when she curled close to his neck as she always did in her early years, perfectly content.

